# Considerations for using minocycline vs doxycycline for treatment of canine heartworm disease

**DOI:** 10.1186/s13071-017-2449-1

**Published:** 2017-11-09

**Authors:** Mark G. Papich

**Affiliations:** Department of Molecular Biomedical Sciences, College of Veterinary Medicine, North Carolina State University, 1060 William Moore Drive, Raleigh, NC 27607 USA

**Keywords:** Minocycline, Doxycycline, *Wolbachia*, Heartworm, Pharmacokinetics, Pharmacodynamics, Monte Carlo simulation

## Abstract

**Background:**

Doxycycline has been considered the first drug of choice for treating *Wolbachia,* a member of the Rickettsiaceae, which has a symbiotic relationship with filarial worms, including heartworms. *Wolbachia*, is susceptible to tetracyclines, which have been used as adjunctive treatments for heartworm disease. Treatment with doxycycline reduces *Wolbachia* numbers in all stages of heartworms and improves outcomes and decreased microfilaremia in dogs treated for heartworm disease. The American Heartworm Society recommends treatment with doxycycline in dogs diagnosed with heartworm disease at a dose of 10 mg/kg twice daily for 28 days. If doxycycline is not available, minocycline can be considered as a substitute. However, minocycline has not undergone an evaluation in dogs with heartworm disease, nor has an effective dose been established. Minocycline is an attractive option because of the higher cost of doxycycline and new pharmacokinetic information for dogs that provides guidance for appropriate dosage regimens to achieve pharmacokinetic-pharmacodynamic (PK-PD) targets.

**Results:**

Published reports from the Anti-*Wolbachia* Consortium (A-WOL) indicate superior in vitro activity of minocycline over doxycycline. Studies performed in mouse models to measure anti-*Wolbachia* activity showed that minocycline was 1.7 times more effective than doxycycline, despite a 3-fold lower pharmacokinetic exposure. To achieve the same exposure as achieved in the mouse infection model, a pharmacokinetic-pharmacodynamic (PK-PD) analysis was conducted to determine optimal dosages for dogs. The analysis showed that an oral minocycline dose of 3.75 to 5 mg/kg administered twice daily would attain similar targets as observed in mice and predicted for human infections.

**Conclusions:**

There are potentially several advantages for use of minocycline in animals. It is well absorbed from oral administration, it has less protein binding than doxycycline (65% vs 92%) allowing for better distribution into tissue, and it is approximately two times more lipophilic than doxycycline, which may result in better intracellular penetration. More work is needed to document efficacy of minocycline for treating canine heartworm disease.

## Background

Tetracyclines are among the oldest antimicrobial agents used in veterinary medicine. This group of antibiotics was discovered in 1944 (chlortetracycline) and later expanded to include the various semisynthetic products that include tetracycline, doxycycline (1967), and minocycline (1972). The tetracycline spectrum of activity is generally broad, with activity against both aerobic and anaerobic gram-positive and gram-negative bacteria, though resistance among these bacteria can be common. Their greatest value, however, lies in the activity against atypical bacteria and some protozoa. The spectrum includes the family Rickettsiaceae, particularly *Rickettsia* and *Ehrlichia* species. Doxycycline has been considered the first drug of choice for these infections.

*Wolbachia* is an obligate intracellular bacteria of the *Rickettsiales* found in canine heartworms. In addition to other therapies, doxycycline has been considered the treatment of choice for canine heartworm infections by the American Heartworm Society (AHS) (https://www.heartwormsociety.org/). This recommendation is based on the observation that filarial worms, including *Dirofilaria immitis*, have a symbiotic relationship with *Wolbachia* spp. [1, 2].

The benefits of anti-*Wolbachia* treatment in heartworm-infected dogs have been well documented [3–7]. The doxycycline administration protocol for dogs is based on the pivotal work by McCall and his colleagues [3–5]. They found that administration of doxycycline to heartworm-infected dogs reduces *Wolbachia* numbers in all stages of heartworms. Doxycycline administration during the first or second month following experimental heartworm infection was lethal to third- and fourth-stage heartworm larvae and decreased microfilaremia [3, 4, 8]. Administration of doxycycline to heartworm-infected dogs appears to inhibit the development of larvae into adult stage worms. The effects of doxycycline are improved when administered in combination with ivermectin [8]. In addition to reducing the viability of *Wolbachia,* doxycycline treatment may reduce the pulmonary inflammatory response caused by *Wolbachia* [3, 6, 7]. Doxycycline treatment reduces *Wolbachia* organisms and their metabolites when the adult worms die, thus reducing the pulmonary vascular reaction [3, 6, 7].

*Wolbachia* organisms have also been implicated as a component in the pathogenesis of other filarial diseases, particularly the filarial worms infecting people in developing countries in tropical regions. These diseases include lymphatic filariasis and onchocerciasis (river blindness). Sharma and colleagues stated that “doxycycline is the current gold standard anti-*Wolbachia* treatment” for these infections in people [9].

The protocol currently suggested by the AHS is to administer doxycycline prior to administration of melarsomine. The current recommendation is oral doxycycline hyclate administered at 10 mg/kg twice daily for 28 days prior to adulticide treatment.

Unfortunately, there are no approved formulations of doxycycline for dogs or cats in the United States. Doxycycline is available in the human formulation as tablets or capsules (doxycycline hyclate). There are some approved formulations available in Europe and other countries for dogs and cats, but these are not allowed in the United States. The cost of human formulations of doxycycline hyclate for veterinary patients has undergone price fluctuations and problems with availability. According to one source, the cost of doxycycline increased over 1000% during one period in the United States (https://www.avma.org/News/JAVMANews/Pages/150115a.aspx), which produced a great burden for pet owners. Alternatives have been considered. One option was to use compounded doxycycline in an oral suspension. But forms of doxycycline hyclate tablets compounded as an oral aqueous suspension for dogs were not stable for longer than 7 days in one study [10]. Other compounded formulations and vehicles were not tested in that study, and it is possible that results could vary if other conditions and excipients were used.

The other alternative considered by veterinarians is minocycline. Although no efficacy studies are available for treating heartworm infections in dogs, it may be an alternative to consider. This paper presents an analysis of existing data, review of the literature to support anti-*Wolbachia* activity of minocycline, a presentation of the pharmacokinetic data available to guide dosing in dogs, and a pharmacokinetic-pharmacodynamic (PK-PD) analysis and Monte Carlo simulations to predict potential activity and dosages for heartworm-infected animals.

### Review of minocycline studies in dogs

Minocycline is available as minocycline hydrochloride tablets and capsules in a generic form for people in 50 and 100 mg sizes. It is not approved for dogs and the use is considered extra-label (but legal). The dosages administered to dogs have not been tested clinically, but based on pharmacokinetic studies and PK-PD analysis, an oral dose of 5 mg/kg twice daily has been suggested for infections caused by *Staphylococcus pseudintermedius* in dogs [11, 12]. Higher doses in dogs have a tendency to produce vomiting.

#### Pharmacokinetic studies

In dogs, three oral dose studies have been published for minocycline [11–13]. These results are summarized in Table 1. Oral absorption is approximately 50%. The terminal half-life is approximately 5 h. The pharmacokinetics for doxycycline also have been performed in dogs [14] and summarized by Maaland et al. [15]. In comparison to minocycline, oral absorption of doxycycline is approximately 60%, with a highly variable half-life of 6 to 29 h.

**Table 1:** Minocycline and doxycycline pharmacokinetics in dogs

Parameter	Doxycycline	Minocycline
Half-life (T-½) (hr)	12.6 (± 11)	5.18 (± 1.02)
VD/F (L/kg)	1.67 (± 0.81)	2.3 (± 0.59)
CL/F (L/kg/h)	0.13 (± 0.06)	0.28 (± 0.08)
Absorption (F)	61% (± 8)	50.3% (± 20.8)
Protein binding %	91–92%	65.8% (in vitro) 50% (in vivo)

*VD/F* volume of distribution per fraction absorbed, *CL/F* clearance per fraction absorbed, *F* fraction absorbed. These parameters are presented as a *weighted mean* value to account for differences in the number of subjects in each study, as well as between-study and within-study variation [11–15]

### Protein binding

Protein binding reduces the amount of active drug because only the unbound fraction of an antibiotic is microbiologically active. In vitro protein binding of minocycline in dogs is 65.8%. In vivo protein binding in dogs (based on in vivo ultrafiltration) is 50% (± 0.17) [11]. For doxycycline, protein binding is much higher: over 90% in dogs [14].

### Effect of feeding

**Fig. 1:**
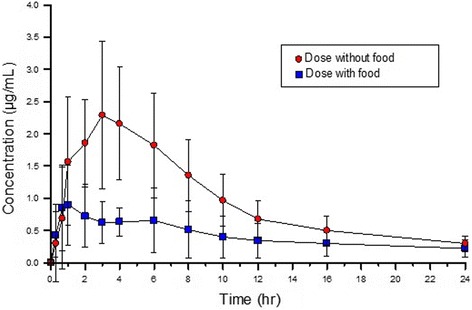
Effect of feeding on oral absorption of minocycline in dogs (approximately 5 mg/kg oral). Data from study by Hnot et al., [12]

Initial studies with minocycline were performed on dogs that had not been fed [11]. Follow-up studies explored the effects of feeding [12]. Shown in Fig. 1 is the significant effect of feeding on oral absorption. The area under the curve (AUC) for the fasted dogs was approximately 1.5-fold higher than the fed dogs [12]. Population pharmacokinetic analysis, using nonlinear mixed effects modeling (NLME, Phoenix software, https://www.certara.com/software/pkpd-modeling-and-simulation/phoenix-nlme/) showed that feeding has a significant effect on the variability of oral absorption in dogs [12].

### Antibacterial activity

Minocycline and doxycycline have broad antibacterial activity that includes gram-positive and gram-negative bacteria, some protozoa, Rickettsiae, and Ehrlichiae. Like other tetracyclines, the mechanism of action of minocycline and doxycycline is to bind to the 30S ribosomal subunit and inhibit protein synthesis.

Interpretive categories (breakpoints) for antibacterial susceptibility testing have been established for minocycline [16] and doxycycline [15, 17]. Because of greater activity, favorable pharmacokinetics, and lower protein binding, minocycline breakpoints are higher than for doxycycline – indicating that some bacteria considered “resistant” to doxycycline may be “susceptible” to minocycline (Table 2).

**Table 2:** Antibacterial interpretive categories (breakpoints) for tetracycline antibacterial agents in dogs from CLSI [17]

Drug	Susceptible	Intermediate	Resistant
Doxycycline	≤ 0.12 μg/mL	0.25 μg/mL	≥ 0.5 μg/mL
Tetracycline	≤0.25 μg/mL	0.5 μg/mL	≥1.0 μg/mL
Minocycline	≤0.5 μg/mL	1.0 μg/mL	≥2.0 μg/mL

### *Anti-*Wolbachia *activity*

Other studies have reported on the important anti-*Wolbachia* activity of minocycline in various models and assays. These studies used a World Health Organization-approved in vitro assay to screen for antifilarial activity. These studies represent the work by the anti-*Wolbachia* Consortium (http://awol.lstmed.ac.uk/), which is an international group of research laboratories and scientists devoted to identifying anti-*Wolbachia* treatments (http://awol.lstmed.ac.uk/). This group has worked to identify other anti-*Wolbachia* treatments as alternatives to doxycycline, which are more effective and can be administered for shorter durations. The assay used is a cell-based assay with a quantitative PCR readout to screen drugs for activity. This 96-well in vitro assay allows for rapid screening of multiple agents. The validation of the assay was described in another paper [18]. In their study, approximately 2600 drugs were screened. Several drugs were identified that had superior activity compared to doxycycline, including other tetracyclines, fluoroquinolones, and rifamycins (rifampin) [18]. The most promising agents were subsequently tested in a nematode-infected mouse model using *Litomosoides sigmodontis,* which is a filarial nematode used in a mouse model for studying filarial infections. In this assay, minocycline treatment significantly reduced the *Wolbachia* load and produced significantly greater response than the equivalent doxycycline treatment. The authors of the study attributed the higher activity of minocycline in this assay to the higher lipophilicity of minocycline over doxycycline [19]. Higher lipophilicity of minocycline may account for higher concentrations of the drug reaching the tissues, such as the nematode hypodermal cords in which the *Wolbachia* organisms reside.

An in vitro study to test antimicrobial agents for anti-*Wolbachia* activity using an *Onchocerca gutturosa* assay in cultured monkey kidney cells was used by Townson and colleagues [20]. *Onchocerca gutturosa* can be used to screen for antifilarial drug activity but is also valid for screening antibiotics for anti-*Wolbachia* activity. In this study, several antibiotics were tested for their anti-*Wolbachia* activity. Although doxycycline showed filaricidal activity, minocycline was more quickly and completely effective, and at lower concentrations. Interestingly, the other tetracycline tested, oxytetracycline, was inactive.

A follow-up study by the anti-*Wolbachia* Consortium examined minocycline as a repurposed anti-*Wolbachia* macrofilaricide [9]. This study tested anti-*Wolbachia* activity of antibiotics in the filarial nematode, *Brugia malayi-*infected mice model. Doxycycline produced greater pharmacokinetic exposure after equivalent doses (higher area under the curve, AUC). However, despite an approximately 3-fold less exposure with minocycline, it was more effective. The PK-PD analysis showed that minocycline is expected to be 1.7 times more effective than doxycycline in people. That study used the mouse data and Monte Carlo simulations to evaluate effective dosages of minocycline for treating filarial infections in people. Here, the same approach is used to predict effective dosages for dogs with heartworm disease.

## Methods

The analysis reported here to predict effective anti-*Wolbachia* minocycline and doxycycline dosages for treating heartworm infections in dogs used similar methods that were employed in the study by Sharma et al. [9] for mice. In the Sharma study [9], pharmacokinetic values from human studies of doxycycline and minocycline were used to derive equivalent dosages for mice. A standard doxycycline or minocycline dose to treat infections in people is either 100 or 200 mg per person per day. From their simulations, researchers concluded that a mouse dose of 25 mg/kg once or twice daily closely emulates the overall daily exposure of a 100 or 200 mg clinical dose of doxycycline and minocycline in people. They used the dosages of these agents derived for mice to test the anti-*Wolbachia* activity in the filarial nematode infection model. The results are shown in Table 3, with 90.35% and 99.5% *Wolbachia* reduction from administration of doxycycline and minocycline, respectively, at a daily dose equivalent to the human dose of 200 mg (100 mg twice daily). The percent reduction in their study [9] was derived from measuring *Wolbachia* loads per female worm compared with untreated controls. For this analysis, an equivalent dose for dogs was derived to obtain the same AUC that would provide similar exposure for the highest efficacy demonstrated in the mouse model.

**Table 3:** Doxycycline and minocycline exposure in mice and humans after administration of standard doses in order to produce similar exposure relationships (AUC) in mice

	100 mg dose Human		200 mg dose Human	
	Doxycycline	Minocycline	Doxycycline	Minocycline
AUC μg·hr./mL	28.4	17.7	56.9	35.4
	Mouse 25 mg/kg once daily		Mouse 25 mg/kg twice daily*	
	Doxycycline	Minocycline	Doxycycline	Minocycline
AUC μg·hr./mL	22.8	7.8	40.6	14.9
*Wolbachia* reduction	66.3%	85%	90.35%	99.5%

*AUC* area under the curve for doxycycline or minocycline exposure after standard dosages. ^*^There were no significant changes in the level of *Wolbachia* depletion when comparing dose escalations between 25 and 80 mg/kg twice daily. Data from Sharma, et al. [9]

### Monte Carlo simulation and probability of target attainment

To calculate the probability of target attainment (PTA), the AUC target was the value with highest efficacy in the mouse model (Table 3). The AUC values used for the Monte Carlo simulation analysis were 7.8 μg⋅hr/mL and 15 μg⋅hr/mL for minocycline, and 22.8 μg⋅hr/mL and 40.6 μg⋅/hr./mL for doxycycline.

To derive the optimum dose to obtain these AUC values, the pharmacokinetic parameters available for dogs were used (Table 1) and entered into Eq. 1.

1 $$ \mathrm{Dose}=\frac{\mathrm{CL}\cdot \mathrm{AUS}}{\mathrm{F}\cdot 24\mathrm{hour}} $$

For the simulations, the clearance value per fraction absorbed was used (CL/F) (Table 1); therefore, fraction absorbed (F) was not used in the calculation. The 24-h term implies that the AUC for a 24-h interval was used. These values were entered into a forecasting program (Crystal Ball, Oracle, version 11.1.2.3.500). Using Crystal Ball, Monte Carlo simulations were generated for 1000 trials, and daily doses ranging from 1.25 to 40 mg/kg were explored (0.625 to 20 mg/kg administered twice daily).

## Results

**Fig. 2:**
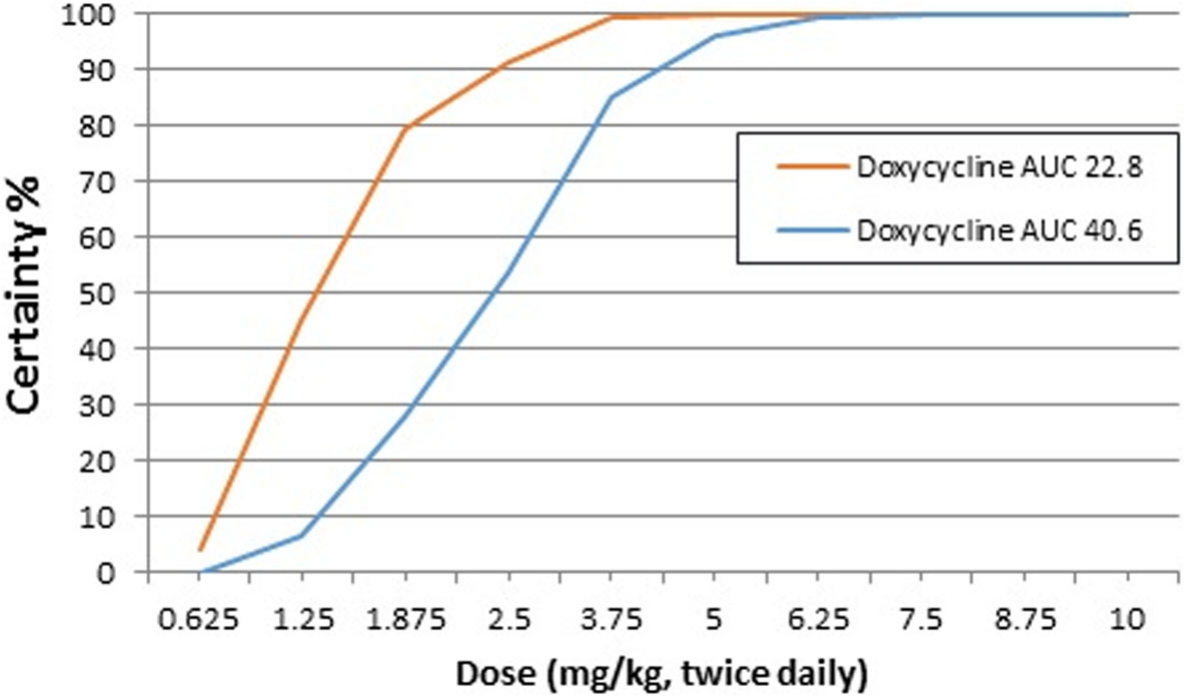
Probability of target attainment (PTA) for doxycycline administered twice daily. Probability of target attainment (certainty) is shown for AUC values of 22.8 μg·hr./mL and 40.6 μg·hr./mL for total (bound and unbound) drug concentration

**Fig. 3:**
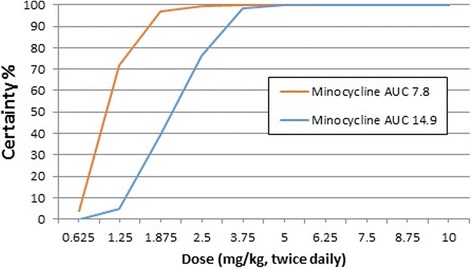
Probability of target attainment (PTA) for minocycline administered twice daily. Probability of target attainment (certainty) is shown for AUC values of 7.8 μg·hr./mL and 14.9 μg·hr./mL for total (bound and unbound) drug concentration

Monte Carlo simulations were used to calculate the PTA (percent certainty) shown in Figs. 2 and 3 and Table 4. Based on this analysis, a 5 mg/kg oral dose twice daily will attain the target for the highest efficacy for doxycycline with >90% certainty. A 3.75 or 5 mg/kg oral dose twice daily for minocycline will attain the target for highest efficacy for minocycline with >90% certainty.

**Table 4:** Probability of target attainment (PTA %) for doxycycline and minocycline at doses ranging from 0.625 to 10 mg/kg administered orally every 12 h to dogs

Drug and AUC Target (μg·hr./mL)	Dose (mg/kg, twice daily, oral)									
	0.625	1.25	1.875	2.5	3.75	5	6.25	7.5	8.75	10
Doxycycline 22.8	4.1	45	79.37	91.5	99.5	99.9	100	100	100	100
Doxycycline 40.6	0.08	6.5	28	53.7	85	96.1	99.63	99.84	100	100
Minocycline 7.8	4	72	97.05	99.6	100	100	100	100	100	100
Minocycline 15	0	4.6	39.3	76	98.3	99.78	100	100	100	100

### Monte Carlo simulation interpretation

Based on the Monte Carlo simulations and data presented in Table 4 and Fig. 2, there is a high probability (>90%) of attaining the highest AUC value associated with efficacy for doxycycline (AUC 40.6 μg⋅hr/mL) with an oral dose of 5 mg/kg twice daily. Based on the same analysis and data presented in Table 4 and Fig. 3, there is a high probability (>90%) of attaining the highest AUC value associated with efficacy for minocycline (AUC 14.9 μg·hr. /mL) with an oral dose of 3.75 to 5 mg/kg twice daily.

## Discussion

Based on this analysis there is a high probability of target attainment (PTA) for treating *Wolbachia* by administering common oral doses of the tetracyclines used in dogs, doxycycline and minocycline. The target concentrations for this analysis were the AUC values for minocycline and doxycycline that were shown to be effective against *Wolbachia* in a mouse model. The doses identified for dogs to attain these targets were 3.75–5 mg/kg twice daily oral minocycline or 5 mg/kg twice daily oral doxycycline. Because 5 mg/kg minocycline twice daily is the most commonly recommended clinical dose for other infections in dogs, this would be an appropriate dose to consider for clinical studies in dogs with heartworm disease. For doxycycline, the dose identified to attain the AUC associated with highest efficacy was 5 mg/kg oral twice daily. This dose is lower than the current AHS recommendation for administration of oral doxycycline to dogs with heartworm disease of 10 mg/kg twice daily for 28 days (https://www.heartwormsociety.org/). Obviously, as seen in Table 3 and Fig. 2, the higher dose of 10 mg/kg twice daily for doxycycline also produces a high certainty of target attainment.

The Monte Carlo simulations performed used the total drug concentrations (both protein bound and unbound fraction) for these tetracyclines. As shown in Table 1 there is considerable plasma protein binding for doxycycline (> 90%) and approximately 50% protein binding for minocycline. Because only the unbound fraction of an antibiotic is microbiologically active, high protein binding can significantly reduce the effective exposure. In the studies reported by Sharma et al. [9], pharmacokinetic parameters and AUC values were calculated for mice used total (bound and unbound) concentrations. Therefore, this analysis for dogs did not account for protein binding. If the AUC is adjusted for free drug, the exposure for doxycycline (free fraction 0.085) would have been much less, and the exposure for minocycline would be reduced by approximately 50% (free fraction 0.50).

This analysis in dogs showed that it may be possible to obtain higher efficacy for treating *Wolbachia* in dogs with minocycline, despite lower exposure. The study by Sharma and colleagues [9] showed that minocycline was 1.7-fold more effective than doxycycline in their mouse model, despite a 3-fold lower exposure than doxycycline. Additional analysis by their group and PK-PD analysis showed that the transfer rate constant to the effect site was 24-fold higher for minocycline in comparison to doxycycline.

Sharma and colleagues [9] analyzed other factors that may explain the higher efficacy for minocycline compared with doxycycline. They performed an analysis of physiochemical properties of doxycycline and minocycline to examine parameters that are associated with permeability across membranes. The lipophilicity (determined by LogP) and number of hydrogen bond donors were superior for minocycline, consistent with higher permeability across membranes. Minocycline also has approximately twice the lipophilicity of doxycycline which is consistent with higher permeability across membranes [19]. Furthermore, minocycline has a molecular polar surface area (PSA) that is aligned with recommended values for permeable compounds. These properties of minocycline, in addition to lower protein binding (higher free fraction available), may have contributed to the greater anti-*Wolbachia* activity in their mouse model.

## Conclusion

This analysis suggests that compared to the “gold standard” doxycycline, minocycline has favorable pharmacokinetics, better activity, higher in vitro activity, and higher in vivo efficacy in mice for treating *Wolbachia* infections. There have been no studies to evaluate efficacy of minocycline for treating heartworm infections in dogs. Pharmacokinetic studies in dogs and the analysis presented here, however, indicate that a dose of 5 mg/kg oral, twice daily (or possibly lower), should be considered for future clinical studies in dogs.

## Abbreviations

AHS: American Heartworm Society
AUC: Area under the curve
hr.: Hour
L/kg: Liters per kilogram
L/kg/hr: Liters per kilogram per hour
PK-PD: Pharmacokinetics/pharmacodynamics
PTA: Probability of target attainment
μg/mL: Micrograms per milliliter
